# Supplementary material for: Brief digital self-care intervention for health anxiety in a Swedish Medical University Clinic: a prospective single-group feasibility study
Source: BMJ Open. 2023 Dec 21;13(12):e077376. doi: 10.1136/bmjopen-2023-077376 (PMC10748877; doi:10.1136/bmjopen-2023-077376)
Supplement: Supplementary data [file bmjopen-2023-077376supp001.pdf]

A feasibility study of a digital intervention for health anxiety

Supplemental material

MEAURES

Anxiety symptoms:

Generalized Anxiety Disorder 7-item scale (GAD-7) (1). The GAD-7 ranges from 0-21 where higher total scores indicate greater severity of anxiety symptoms.

Depression:

Patient Health Questionnaire 9 (PHQ-9) (2). The PHQ-9 ranges from 0 to 27 where higher total scores indicate greater severity of depression.

Quality of life:

Brunnsviken Brief Quality of Life Questionnaire (BBQ) (3). The BBQ is a 12-item questionnaire covering six different life domains: leisure time, view on life, creativity, learning, friends and friendship, and view of self. Scores range from 0 to 96, where higher values indicate higher quality of life.

Additional adherence measures:

Subjective adherence was determined by how much they had worked with ERP measured by a weekly questionnaire. In this questionnaire, participants were instructed to rate whether they had worked with ERP *not at all, occasionally, a majority of the days* or *every day* during the previous week.

Adherence was also explored by the number of different types of exposure exercises performed by participants measure by a questionnaire where participants at the end of the program was instructed to answer yes or no to whether they engaged in each component (type of exposure exercise) of the intervention.

Usability:

To assess usability, The System Usability Scale (SUS) was used (4). The SUS consists of ten items, with a range of 0-4 points per item. The items are summed up and calculated with a formula resulting in a total score between 0 and 100, with a higher score reflecting better system usability.

Experience with the program:

Experience with the self-care intervention was evaluated through a non-validated questionnaire. The questionnaire comprised four questions where participants were asked whether the intervention was perceived as likable, easy to understand, whether examples given felt relevant and whether functionality and information contributed to the participant feeling overwhelmed. The questions were answered on a four-point scale from 0 (strongly disagree) to 3 (strongly agree), with response alternatives reversed for the question regarding feeling overwhelmed. A score above 1.5 indicates positive attitudes.

RESULTS

Table e1 presents means, standard deviations and effect sizes the GAD-7, PHQ-9 and BBQ over the treatment- and follow-up period. At 3 month-follow-up, 14 out of 19 interviewed participants (74%) did not longer fulfil diagnostic criteria for IAD measured with the HPDI.

Experience with the self-care intervention:

Österman S, *et al. BMJ Open* 2024; 13:e077376. doi: 10.1136/bmjopen-2023-077376

A feasibility study of a digital intervention for health anxiety

The participants' responses on the study-specific questions on usefulness at post-treatment suggest that they found the intervention easy to understand (M 2.1, SD 0.72 CI 1.76-2.44) and as having relevant clinical examples (M 2.1, SD 0.83 CI 1.66-2.44). The scores on questions about the intervention being perceived as enjoyable (M 1.6, SD 0.82) and not overwhelming (M 1.75, SD 0.72 CI 1.22- 1.98) were somewhat lower.

Adherence:

In any given week during the program, 48-72% of participants reported working with ERP occasional days and 16-44 % reported working ERP a majority of the days. Moreover, 92 % of the participants reported working with ERP at least one week during the program. The mean weeks of active work with ERP was 4.6 out of 8. At post-treatment, 75 % of the participants reported that they had worked with and benefited from interoceptive exposure, 85 % from response prevention, 85 % from exposure in vivo and 70 % from imaginary exposure.

Table S1. Secondary results

Measure	Time point				Changes from PRE	
		N	M	SD	Within-group effect size	Confidence interval
GAD-7	PRE	25	8.76	4.86	NA	NA
	POST	20	5.4	4.56	0.70	0.09-1.30
	FU	22	4	3.73	1.07	0.46-1.68
PHQ-9	PRE	25	6.56	6.10	NA	NA
	POST	20	3.65	3.65	0.56	-0.04-1.15
	FU	22	3.45	4.24	0.58	-0.01-1.16
BBQ	PRE	25	52.16	19.20	NA	NA
	POST	20	58.7	17.45	0.35	-0.24-0.94
	FU	22	61.86	22.40	0.46	-0.12-1.04
SUS	Post		75.1	14.9	NA	NA

Note. Abbreviations: *GAD-7:* Generalized Anxiety Disorder 7-item scale; *PHQ-9* Patient Health Questionnaire - 9 Item; *BBQ:* Brunnsviken Brief Quality of Life Questionnaire; *SUS:* The System Usability Scale; *HPDI*: The Health Preoccupation Diagnostic Interview. *FU:* Follow up, refers to 3 months after post-treatment.

A feasibility study of a digital intervention for health anxiety

Supplementary references:

1. Spitzer RL, Kroenke K, Williams JBW, Löwe B. A Brief Measure for Assessing Generalized Anxiety Disorder: The GAD-7. Archives of Internal Medicine. 2006;166(10):1092-7.
2. Kroenke K, Spitzer RL, Williams JB. The PHQ-9: validity of a brief depression severity measure. J Gen Intern Med. 2001;16(9):606-13.
3. Lindner P, Frykheden O, Forsström D, Andersson E, Ljótsson B, Hedman E, et al. The Brunnsviken Brief Quality of Life Scale (BBQ): Development and Psychometric Evaluation. Cognitive behaviour therapy. 2016;45(3):182-95.
4. Bangor A, Kortum PT, Miller JT. An Empirical Evaluation of the System Usability Scale. International Journal of Human–Computer Interaction. 2008;24(6):574-94.
