# Supplementary material for: Brief digital self-care intervention for health anxiety in a Swedish Medical University Clinic: a prospective single-group feasibility study
Source: BMJ Open. 2023 Dec 21;13(12):e077376. doi: 10.1136/bmjopen-2023-077376 (PMC10748877; doi:10.1136/bmjopen-2023-077376)
Supplement: Author's manuscript [file bmjopen-2023-077376.draft_revisions.pdf]

BMJ Open is committed to open peer review. As part of this commitment we make the peer review history of every article we publish publicly available.

When an article is published we post the peer reviewers' comments and the authors' responses online. We also post the versions of the paper that were used during peer review. These are the versions that the peer review comments apply to.

The versions of the paper that follow are the versions that were submitted during the peer review process. They are not the versions of record or the final published versions. They should not be cited or distributed as the published version of this manuscript.

BMJ Open is an open access journal and the full, final, typeset and author-corrected version of record of the manuscript is available on our site with no access controls, subscription charges or pay-per-view fees (http://bmjopen.bmj.com).

If you have any questions on BMJ Open's open peer review process please email info.bmjopen@bmj.com

BMJ Open

A feasibility study of a digital self-care intervention for health anxiety: maintaining quality and safety while reducing patient burden and strain on clinical resources, compared to a more comprehensive intervention

Journal:	*BMJ Open*
Manuscript ID	bmjopen-2023-077376
Article Type:	Original research
Date Submitted by the Author:	03-Jul-2023
Complete List of Authors:	Österman, Susanna; Karolinska Institute, Department of clinical neuroscience<br>Hentati, Amira; Karolinska Institute, Department of clinical neuroscience<br>Forsell, Erik; Karolinska Institute, Department of clinical neuroscience<br>Axelsson, Erland; Karolinska Institute, Division of Family Medicine and Primary Care, Department of Neurobiology, Care Sciences and Society<br>Hedman-Lagerlöf, Erik ; Karolinska Institute, Department of clinical neuroscience<br>Lindefors, Nils; Karolinska Institute, Department of clinical neuroscience<br>Ivanov, Volen; Karolinska Institute, Department of clinical neuroscience<br>Kraepelien, Martin; Karolinska Institute, Department of clinical neuroscience
Keywords:	MENTAL HEALTH, PSYCHIATRY, Anxiety disorders < PSYCHIATRY

SCHOLARONE™
Manuscripts

I, the Submitting Author has the right to grant and does grant on behalf of all authors of the Work (as defined in the below author licence), an exclusive licence and/or a non-exclusive licence for contributions from authors who are: i) UK Crown employees; ii) where BMJ has agreed a CC-BY licence shall apply, and/or iii) in accordance with the terms applicable for US Federal Government officers or employees acting as part of their official duties; on a worldwide, perpetual, irrevocable, royalty-free basis to BMJ Publishing Group Ltd ("BMJ") its licensees and where the relevant Journal is co-owned by BMJ to the co-owners of the Journal, to publish the Work in this journal and any other BMJ products and to exploit all rights, as set out in our licence.

The Submitting Author accepts and understands that any supply made under these terms is made by BMJ to the Submitting Author unless you are acting as an employee on behalf of your employer or a postgraduate student of an affiliated institution which is paying any applicable article publishing charge ("APC") for Open Access articles. Where the Submitting Author wishes to make the Work available on an Open Access basis (and intends to pay the relevant APC), the terms of reuse of such Open Access shall be governed by a Creative Commons licence – details of these licences and which Creative Commons licence will apply to this Work are set out in our licence referred to above.

Other than as permitted in any relevant BMJ Author's Self Archiving Policies, I confirm this Work has not been accepted for publication elsewhere, is not being considered for publication elsewhere and does not duplicate material already published. I confirm all authors consent to publication of this Work and authorise the granting of this licence.

For peer review only

A feasibility study of a digital intervention for health anxiety

A feasibility study of a digital self-care intervention for health anxiety: maintaining quality and safety while reducing patient burden and strain on clinical resources, compared to a more comprehensive intervention

Word count (main text): 2428

Key words: Health anxiety; illness anxiety disorder; somatic symptom disorder, cognitive behavioral therapy; digital intervention; self-care; self-guided

For peer review only

A feasibility study of a digital intervention for health anxiety

Abstract

Objectives:

In routine psychiatric care in Stockholm, Sweden, a comprehensive therapist-guided intervention for clinically significant health anxiety is implemented. However, there is a need for more easily accessible self-care interventions to improve treatment dissemination. This study aimed to transform an existing therapist-guided digital intervention into a self-care intervention, reducing patient burden and clinical resources while maintaining quality and safety.

Design:

An uncontrolled feasibility study

Setting:

A medical university in Stockholm, Sweden

Participants:

Twenty-five adults utilized the self-care intervention and underwent telephone assessments, along with completing self-rated questionnaires.

Intervention:

The newly developed 8-week self-care intervention was designed to be user-friendly without therapist guidance, and to facilitate high levels of behavioural engagement.

Primary and secondary outcome measures:

Indicators of quality and safety, including changes in health anxiety severity (primary), clinician time, participant adherence, perceived credibility/satisfaction with the intervention

For peer review only - http://bmjopen.bmj.com/site/about/guidelines.xhtml

and adverse events, were benchmarked against a previous study of the more comprehensive intervention it was based on.

Results:

Compared to the original intervention, the self-care intervention was condensed in terms of text (up to 70% less reading), duration (8 weeks instead of 12), and number of exercises. Quality indicators were similar to the original version. A majority of participants worked actively with core components in the self-care intervention. Preliminary within-group analysis showed promising effects on health anxiety from pre-treatment to the 3-month follow-up (g = 1.37; 95% CI, 0.74-2.00). No serious adverse events were reported.

Conclusions:

This brief digital self-care intervention shows potential for increasing access to treatment for individuals with health anxiety, while reducing burden on patients and clinical resources. Future studies should investigate the optimal type of intervention and support for different individuals, and if non-inferiority can be established.

Trial registration:

ClinicalTrials.gov (NCT05446766)

For peer review only - http://bmjopen.bmj.com/site/about/guidelines.xhtml

For peer review only

A feasibility study of a digital intervention for health anxiety

Strengths and limitations of this study

- Uncontrolled feasibility study
- A self-recruited volunteer sample
- Results were benchmarked against a previously published randomised trial

For peer review only

A feasibility study of a digital intervention for health anxiety

INTRODUCTION

Clinically significant health anxiety is a psychiatric condition characterized by an excessive fear of, or preoccupation with, having or developing a serious medical illness (1). Individuals who suffer from clinically significant health anxiety tend to report high distress, disability and increased health service utilization (2). Therefore, the development of accessible, evidence-based interventions for this debilitating condition is a major challenge for health care today.

Clinically significant health anxiety can be effectively treated with therapist-guided internet-based cognitive behavioural therapy (ICBT) (3), a format which requires less therapist support per patient compared to traditional face-to-face Cognitive Behavioral Therapy (CBT), thus making the treatment more cost effective (4-6). In order to further increase the clinical utility of CBT, the development of programs without therapist support, i.e. self-guided or self-care interventions, represent an attractive avenue because of their more immediate scalability. Self-care interventions may also reduce stigma, thereby decreasing barriers to seeking treatment (7). Furthermore, data indicate that many individuals with severe health anxiety would prefer working with self-guided interventions over guided versions (8).

A recurrent finding in research highlights the challenge of maintaining comparable quality in self-help interventions, similar to that of guided interventions (9). However, there are promising studies suggesting that self-care interventions can be developed to a level where they can be comparable to guided ones (10). Several features have been cited as important in order to maintain effectiveness when clinician guidance is removed (11). These include a clinical interview prior to and post-intervention, monitoring of participant progress, high quality treatment material and automated features such as automatic messages prompting and reinforcing participants' work with the intervention

In Sweden, a 12-week guided exposure-based digital intervention for health anxiety (12-14) has been successfully implemented in routine psychiatric care at the Stockholm Internet

For peer review only - http://bmjopen.bmj.com/site/about/guidelines.xhtml

psychiatry unit since 2018 (https://www.internetpsykiatri.se/en/). Exposure-based therapy, comprising the key component *exposure with response prevention* (ERP), involves gradual exposure to feared health-related situations or stimuli while refraining from compulsive health-related behaviours (15).

To further meet patient needs, enhance treatment accessibility, and reduce the time and costs associated with clinician support, this comprehensive digital intervention could potentially be offered as a self-help intervention. The guided 12 week-digital intervention has, in addition to being used in a guided format, previously been in a self-guided but otherwise unaltered version (14). The self-guided version showed clinically significant effects but somewhat lower adherence compared to the guided intervention (around 50 % completed modules compared to 70 %). In both the guided and self-guided version of the 12-week long intervention, the key component ERP was not introduced before week 4-5 in the treatment program. This means that many individuals with low treatment adherence, especially in the unguided self-guided version, did not have sufficient time to work with this key component. Other drawbacks of the original intervention include its relative comprehensiveness, which places significant demands on the patient, and the lack of optimization in terms of user-friendliness or suitability for self-guided use.

The aim of the present study was to develop a brief self-care intervention for individuals with clinically significant health anxiety with the potential to improve scalability and decrease participant burden and clinician resources while maintaining quality. The aim was further to benchmark the new self-care intervention against a previous study of the more extensive and resource-demanding therapist guided intervention it was based on (14).

For peer review only - http://bmjopen.bmj.com/site/about/guidelines.xhtml

MATERIAL AND METHODS

Context and design

This was a prospective single-group feasibility study based at Karolinska Institutet, Stockholm, Sweden. The study was preregistered on ClinicalTrials.gov (NCT05446766). Indicators of quality and safety was benchmarked against a more comprehensive digital intervention, in both guided and unguided versions from a previously published randomised trial (14).

Patient and Public Involvement

Patients were not involved in the design of the study or the intervention.

Participants and recruitment

In order to provide sufficiently reliable estimates of quality and safety, a sample of 20 participants was deemed sufficient. To allow for some attrition, the goal was to recruit 25 participants. These were recruited through advertisements in social media under the heading "Do you worry a lot about your health?" and started the intervention after a clinical interview via telephone (average time: 40 minutes, SD: 6.9). This assessment was conducted by a clinical psychologist, and included the Health Preoccupation Diagnostic Interview (HPDI) (16) and the Mini-International Neuropsychiatric Interview (MINI) (17). Participants also underwent an interview via telephone post intervention (average time: 23 minutes, SD: 5.7). Inclusion criteria were 1) age of 18 years or older, 2) a principal diagnosis of illness anxiety disorder or somatic symptom disorder (the prototypical diagnoses for clinically significant health anxiety according to the DSM-5) (1, 18), 3) currently not receiving similar psychological treatment for health anxiety, and 4) no serious medical illness or urgent need for more intensive psychiatric care. If participants were on psychotropic medication, the dose

For peer review only

had to be stable for 4 weeks prior to the baseline assessment. Recruitment started on September 6, 2022 and the last follow-up was collected on March, 29, 2023.

Intervention

The digital self-care intervention was administered on a secure web platform with interface that mimicked a smartphone application. This optimized user interface was developed together with experts in user experience with the aim of being easy to use without therapist guidance and in order to facilitate high levels of behavioural engagement (19). Participants also received automated text messages in order to encourage engagement with the treatment. If in need of technical support, they could ask for assistance via the platform and were then contacted via telephone or email within approximately 1-3 days. For a flow chart of the clinical procedures, se Figure 1.

The main therapeutic focus was ERP, which was introduced the very first week of the intervention. ERP is thought to decrease symptoms of health anxiety through repeated confrontation with feared or avoided stimuli (e.g., body sensations, situations) while refraining from engaging in maladaptive health-related behaviors (e.g., body checking) (15). The intervention included both mandatory and optional text sections with the latter mainly consisting of different clinical vignettes in order to make the intervention more personalized. See Table 1 for a detailed comparison between the new self-care intervention and the original comprehensive digital intervention in both therapist-guided and self-guided guided versions. See Figure 2 for comparative screenshots.

Table 1. Overview of the themes of the original and the new interventions including word count

For peer review only - http://bmjopen.bmj.com/site/about/guidelines.xhtml

A feasibility study of a digital intervention for health anxiety

	**Original digital intervention (same content in guided and self-guided version)**		**New digital self-care intervention**		
Week	Content	Word length (*n*)	Content	Word length (*n*) Mandatory	Word length (*n*) Optional
1	Introduction to CBT and health anxiety. How to navigate the program. Introduction to mindfulness and behavior diary.	8528	Information about health anxiety. How to navigate the program. Psychoeducation about ERP	5857	2493
2	Presentation of the CBT model of severe health anxiety, continued mindfulness training and behavior diary.	6558	The role of bodily symptoms. Exposure: interoceptive exposure.	2564	1248
3	Cognitive processes, continued mindfulness training.	7699	The role of safety behaviors and the importance of response prevention. Participants continue to practice ERP.	2878	3185
4	Introduction to exposure: interoceptive exposure exercises, continued mindfulness training	7961	Avoidance behavior and exposure to health anxiety provoking stimuli (In vivo exposure). Participants continue to practice ERP	1610	3059
5	Response prevention, continued mindfulness training. Participants continue to practice ERP	7454	Thoughts: Exposure to illness thoughts through imaginal exposure. Participants continue to practice ERP	2120	4730
6	Exposure to health anxiety provoking stimuli (exposure in vivo). Participants continue to practice ERP	5702	Thoughts: Exposure to illness thoughts through imaginal exposure. Participants continue to practice ERP	1418	4404

For peer review only - http://bmjopen.bmj.com/site/about/guidelines.xhtml

A feasibility study of a digital intervention for health anxiety

7	Imaginal exposure and exposure to the fear of death. Participants continue to practice ERP	5381	Exposure and quality of life. Participants continue to practice ERP	1555	3748
8	Continued imaginal exposure. Participants continue to practice ERP	3486	Relapse prevention: construction of a relapse plan and plan for future goals. How to handle health-care utilization in the future.	1845	2066
9	Common obstacles to exposure. Participants continue to practice ERP	1504	NA		
10	Setting new goals. Participants continue to practice ERP	396	NA		
11	A summary of the treatment. Values and quality of life. Participants continue to practice ERP	3197	NA		
12	Maintaining gains and relapse prevention. How to handle health-care utilization in the future.	3522	NA		
Total		61388		19847	24933

Abbreviations: CBT Cognitive behaviour therapy*, ERP* Exposure with response prevention, *NA* Not applicable. Original digital intervention refers to the intervention described in Hedman et al (2016)(14)

[INSERT FIGURE 1 HERE]

[INSERT FIGURE 2 HERE]

Measures

The Health Preoccupation Diagnostic Interview (HPDI)(16) is a structured interview designed to aid the assessment of illness anxiety disorder and somatic symptom disorder according to DSM-5 criteria. The HPDI was administered via telephone pre-treatment and at 12-week follow-up.

For peer review only - http://bmjopen.bmj.com/site/about/guidelines.xhtml

Self-report questionnaires were administered on the same technical platform where the self-care intervention was delivered and participants received text reminders about these. Primary outcome was change in health anxiety as measured with the widely used 14-item Short Health Anxiety Inventory (SHAI-14) which has excellent psychometric properties (20) and shows evidence of good diagnostic utility in both clinical and non-clinical settings (21). Participants completed the SHAI-14 at baseline, each week during treatment, after treatment (week 8), after four additional weeks (week 12) and at the 3-month follow-up (week 24).

To assess credibility and expectancy, the five-item version of Credibility/Expectancy Questionnaire (CEQ) was administered 2 weeks after treatment start (22). A total score of 30 points or higher is considered to indicate adequate treatment credibility. At post-treatment, satisfaction with the treatment was evaluated using the Client Satisfaction Questionnaire (CSQ-8) (23). To assess safety, information on possible adverse events were collected with an open-ended question at post-treatment where participants reported whether they had experienced any negative or unwanted effects of the treatment. Adherence was determined in terms of the number of completed modules, and also through weekly questions about use of ERP. Additional measurements of psychiatric symptoms, quality of life and adherence are described in Supplement 1.

Analysis

Most quality indicators, including adherence, patient feedback and symptom outcomes, are reported descriptively. All analyses were based on observed data. Within-group standardized effect sizes were calculated for symptom change (Hedges *g* based on pooled SD with 95% confidence interval [CI]) at week 12 and at the 3-months follow-up. Week 12 was chosen to be able to compare the within-group effect sizes with the 12 week long interventions in the reference study (14). This comparison was with previously published data and was intended

For peer review only - http://bmjopen.bmj.com/site/about/guidelines.xhtml

as a non-randomized benchmark. Remission was defined as scoring below 18 points on the SHAI-14 (21).

Ethical considerations

The new self-care intervention was developed based on a previously evaluated treatment protocol which has been deemed safe. The authors declare that all procedures contributing to this work comply with the ethical standards of the relevant national and institutional committees on human experimentation and with the Helsinki Declaration of 1975, as revised in 2008. All procedures involving human subjects/patients were approved by the ETHICAL BOARD (INFORMATION BLINDED).

RESULTS

In total, 25 out of 33 interviewed participants were included and received access to the self-care intervention. Twenty-two participants (88%) provided outcome data at the week 12 follow-up, and 23 (92%) did so at the 3-month follow-up. Overall, participant demographics were comparable to those in the reference study. For participant demographics, revisions of the intervention and comparisons of quality indicators between the new self-care intervention and the reference study, see Table 2. For an overview on the proportions of participant who scored below 18 points on the SHAI-14 (criteria for remission) between time points, see Figure 3. All participants except two (23/25, 92 %) reported having worked with ERP during the treatment period. Regarding safety, three participants of those who answered the questions on adverse events (3/22, 14 %) experienced an adverse event during the treatment period. These were (i) increased anxiety, (ii) increased awareness of bodily symptoms and (iii) feelings of being alone/exposed and not being able to discuss this with a therapist. There were no reports of serious adverse events. See Supplement 1 for outcomes on additional secondary measures.

For peer review only - http://bmjopen.bmj.com/site/about/guidelines.xhtml

A feasibility study of a digital intervention for health anxiety

Table 2. Participant demographics, program characteristics and quality indicators benchmarked against the original interventions in the reference study

Part of treatment	Original digital intervention (guided version)	Original digital intervention (self-guided version)	New digital self-care intervention
**Demographics**			
	(*n*=32)	(*n*=33)	(*n*=25)
Gender, *n* (%)	22 (88%) female	24 (73%) female	21 (84%) female
Age<br>Mean age (SD)<br>Minimum-maximum	 38.6 (12.6)<br>22-70	 37.4 (11.6)<br>20-72	 41.0 (15.2)<br>21-70
Occupational status, *n* (%)<br>Working full-time/part-time<br>Retired, on sick-leave or unemployed<br>Other	 22 (69%)<br>3 (9%)<br>7 (22%)	 27 (82%)<br>3 (9%)<br>4 (12%)	 15 (60%)<br>7 (28%)<br>8 (32%)
Marital status<br>Married or de facto, *n* (%)	 28 (88%)	 29 (88%)	 17 (68%)
Years with severe health anxiety mean (SD)	7.2 (5.7)	7.8 (6.4)	9.0 (9.4)
Psychiatric comorbidity [a]	13 (41%)	12 (36%)	14 (56 %)
Stabilised psychotropic medication, SSRIs or SNRIs, *n* (%)	7 (22%)	7 (21%)	8 (32%)
Severity at pre SHAI-14 (0-42)	26.8 (5.4)	28.2 (6.1)	25.7 (6.1)
**Program**			
Duration	12 weeks	12 weeks	8 weeks
User interface	Desktop optimized	Desktop optimized	Mobile optimized
Number of words	60426	60426	Mandatory: 19847<br>Optional: 24933
Therapist time	M (SD)= 63.6 (35.4) minutes/participant	0 minutes by design	0 minutes by design
Means of support	Personalized therapist messages	None	Generalized automated text-messages
Inactivity reminders	Text-message or phone call from clinician	Text-message or phone call from clinician	Generalized automated text-messages

For peer review only - http://bmjopen.bmj.com/site/about/guidelines.xhtml

A feasibility study of a digital intervention for health anxiety

Unlocking new modules	Unlocked from start with instruction to complete them in consecutive order	Unlocked from start with instruction to complete them in consecutive order	Automated if/when: 1 week had passed + participant interacted with all content
**Quality indicators**			
Adherence, modules M (SD), %	8.6 (3.0), 72 %	6.6 (3.6), 55 %	5.3 (2.8), 66 %
Treatment credibility M (SD)	37.1 (6.9)	36.8 (7.9)	33.5 (13.4)
Completed 12 week quality indicators, *n* (%)	31 (97%)	32 (97%)	22 (88%)
Effect size (95% CI) Within-group effect on primary outcome 12 weeks [b]	1.55 (0.87-2.22)	1.31 (0.84-1.78)	1.42 (0.78-2.06)
Below cut-off (remission) at 12 weeks [b], *n* (%)	19 (59%)	17 (52 %)	14 (64%)
Treatment satisfaction M (SD)	26.4 (4.6)	24.8 (4.1)	23.6 (5.3)
Adverse events (at least one) *n* (%)	6 (19%)	4 (12%)	3 (14%)
Completed FU quality indicators, *n* (%)	31 (97%)	27 (82%)	23 (92%)
Effect size (95% CI) Within-group effect on primary outcome FU [c]	2.23 (1.31-3.14)	1.52 (0.90-2.15)	1.37 (0.74-2.00)
Below cut-off Remission at FU [c], *n* (%)	20 (63 %)	17 (52%)	12 (52%)

Note [a] Psychiatric comorbidity refers to having a current DSM-5 diagnosis according to the M.I.N.I **(17)** [b] 12 week refers to post treatment in the reference study and 4 weeks after post treatment in the new intervention. [c] Follow-up period (FU) refers to 6 months after post-treatment in the reference study and 3 months after post-treatment in the new intervention. Remission is defined as a score below 18 points on primary outcome. *Abbreviations: M=* Mean, *SD*=Standard deviation, *FU*= Follow-up. *SHAI-14*=14 item Short health anxiety inventory. Original digital intervention refers to the intervention described in Hedman et al (2016) (14)

[INSERT FIGURE 3 HERE]

For peer review only - http://bmjopen.bmj.com/site/about/guidelines.xhtml

DISCUSSION

The aim of this study was to develop and evaluate the quality of a newly developed brief digital self-care intervention for individuals with clinically significant health anxiety and compare it to the more comprehensive and resource-demanding digital intervention it was based on. The purpose of this was to further enhance treatment accessibility for a patient population that currently presents a challenge to the healthcare system. In comparison to both the therapist-guided and self-guided version of the original intervention in the reference study, quality indicators such as preliminary within-group effect sizes, participant adherence, satisfaction with treatment as well as safety were promising. Importantly, almost all of the participants in the new self-care intervention worked with ERP during the treatment period.

Compared to the reference study, the participants appear to share similar demographics and disease burden and the time spent on interviews of around 60 minutes per participant was also similar (14). However, in the new self-care intervention, the overall clinician workload was reduced since no written guidance were provided which was the case in the guided original intervention where clinician on average posted 18.4 text messages (SD 9.6), mean time: 63.6 minutes (SD: 35.4). Moreover, in both the guided and the self-guided original intervention, the clinician contacted the participant via SMS or phone if they had not been active on the platform for a week, which was not the case in the new self-care intervention. This moderately high level of structure and contact may have worked as a form of engagement enhancing procedures - similar to written guidance. The relatively high level of adherence observed in the new self-care intervention, despite the absence of such contact, is therefore promising and aligns with previous studies indicating that a well-designed self-help intervention may compensate for the lack of therapist support (11, 24). Furthermore, preliminary within-group effects seemed promising given the fact that the new self-care intervention posed less burden on participants due to a shorter treatment length, less and more

For peer review only - http://bmjopen.bmj.com/site/about/guidelines.xhtml

simplified text and a significant reduction in the number of different types of mandatory exercises. Since the guided intervention in the reference study had seemingly higher remission rates at the follow-up assessment, there may, however, be some participants who would benefit from receiving additional guidance. Future studies could help identify who would benefit from such guidance.

Although preliminary, this study supports the notion that a brief digital self-care intervention is feasible and can be comparable to more comprehensive guided versions and should be further examined in larger studies. In order to maintain adherence as well as treatment effects, factors such as the self-care intervention being provided within a clinical context with a short clinical interview prior and post to the intervention are potentially important.

The study has some limitations such as its small sample size and the absence of randomization. Since the comparisons with the original comprehensive intervention in the reference study were not randomized, there was some apparent differences in population characteristics, such as levels of psychiatric comorbidity. This renders caution in all interpretation of differences and similarities in the comparison of interventions. Further, the sample consisted of self-recruited volunteers. However, if implemented, a brief self-care intervention such as the current one may be a means to reach individuals who prefer working on their own and/or who would not otherwise seek help due to stigma or other reasons. It could also possibly serve as a first low intensity intervention in a stepped care model. Additionally, the vast majority of patients receiving ICBT in implemented regular care settings are also self-referred (25).

In conclusion, the quality indicators did not seem to show any substantial differences between the brief self-care intervention compared to the original more resource demanding intervention. This highlights the need for larger randomized studies evaluating what type of intervention works for whom, the role of guidance as well as the level of comprehensiveness

For peer review only

from both a cost-effectiveness as well as participant- and clinician burden perspective, as well as studies of possible non-inferiority. If this brief self-care intervention is found to be both effective and resource effective, it could be a highly useful treatment option for patients with clinically significant health anxiety.

Funding

This study was supported by the Swedish Ministry of Health and Social affairs (grant number S2018/03855/FS). The ministry had no influence on study design, data collection, analysis or publication.

For peer review only

For peer review only - http://bmjopen.bmj.com/site/about/guidelines.xhtml

A feasibility study of a digital intervention for health anxiety

References:

1. Axelsson E, Hedman-Lagerlöf E. Validity and clinical utility of distinguishing between DSM-5 somatic symptom disorder and illness anxiety disorder in pathological health anxiety: Should we close the chapter? J Psychosomat Res. 2023;165:111133.
2. Fink P, Ørnbøl E, Christensen KS. The outcome of health anxiety in primary care. A two-year follow-up study on health care costs and self-rated health. PloS one. 2010;5(3):e9873.
3. Axelsson E, Hedman-Lagerlöf E. Cognitive behavior therapy for health anxiety: systematic review and meta-analysis of clinical efficacy and health economic outcomes. Expert review of pharmacoeconomics & outcomes research. 2019;19(6):663-76.
4. Hedman E, Andersson E, Ljotsson B, Axelsson E, Lekander M. Cost effectiveness of internet-based cognitive behaviour therapy and behavioural stress management for severe health anxiety. BMJ open. 2016;6(4):e009327.
5. Axelsson E, Andersson E, Ljótsson B, Björkander D, Hedman-Lagerlöf M, Hedman-Lagerlöf E. Effect of Internet vs Face-to-Face Cognitive Behavior Therapy for Health Anxiety: A Randomized Noninferiority Clinical Trial. JAMA Psychiatry. 2020;77(9):915-24.
6. Ophuis RH, Lokkerbol J, Heemskerk SCM, van Balkom AJLM, Hiligsmann M, Evers SMAA. Cost-Effectiveness of Interventions for Treating Anxiety Disorders: A Systematic Review. Journal of affective disorders. 2017;210:1-13.
7. Mills H, Mulfinger N, Raeder S, Rüsch N, Clements H, Scior K. Self-help interventions to reduce self-stigma in people with mental health problems: A systematic literature review. Psychiatry Research. 2020;284:112702.
8. Newby JM, Haskelberg H, Hobbs MJ, Mahoney AEJ, Mason E, Andrews G. The effectiveness of internet-delivered cognitive behavioural therapy for health anxiety in routine care. Journal of Affective Disorders. 2020;264:535-42.
9. Baumeister H, Reichler L, Munzinger M, Lin J. The impact of guidance on Internet-based mental health interventions—A systematic review. Internet interventions. 2014;1(4):205-15.
10. Domhardt M, Geßlein H, von Rezori RE, Baumeister H. Internet- and mobile-based interventions for anxiety disorders: A meta-analytic review of intervention components. Depression and Anxiety. 2019;36(3):213-24.
11. Titov N, Dear BF, Staples LG, Terides MD, Karin E, Sheehan J, et al. Disorder-specific versus transdiagnostic and clinician-guided versus self-guided treatment for major depressive disorder and comorbid anxiety disorders: A randomized controlled trial. Journal of anxiety disorders. 2015;35:88-102.
12. Hedman E, Andersson G, Andersson E, Ljotsson B, Ruck C, Asmundson GJ, et al. Internet-based cognitive-behavioural therapy for severe health anxiety: randomised controlled trial. The British journal of psychiatry : the journal of mental science. 2011;198(3):230-6.
13. Hedman E, Axelsson E, Gorling A, Ritzman C, Ronnheden M, El Alaoui S, et al. Internet-delivered exposure-based cognitive-behavioural therapy and behavioural stress management for severe health anxiety: randomised controlled trial. The British journal of psychiatry : the journal of mental science. 2014;205(4):307-14.
14. Hedman E, Axelsson E, Andersson E, Lekander M, Ljótsson B. Exposure-based cognitive-behavioural therapy via the internet and as bibliotherapy for somatic symptom disorder and illness anxiety disorder: randomised controlled trial. The British journal of psychiatry : the journal of mental science. 2016;209(5):407-13.
15. Visser S, Bouman TK. The treatment of hypochondriasis: exposure plus response prevention vs cognitive therapy. Behav Res Ther. 2001;39(4):423-42.
16. Axelsson E, Andersson E, Ljotsson B, Wallhed Finn D, Hedman E. The health preoccupation diagnostic interview: inter-rater reliability of a structured interview for

For peer review only - http://bmjopen.bmj.com/site/about/guidelines.xhtml

diagnostic assessment of DSM-5 somatic symptom disorder and illness anxiety disorder. Cognitive behaviour therapy. 2016;45(4):259-69.
17. Lecrubier Y, Sheehan D, Hergueta T, Weiller E. SEC55-1 - The mini international neuropsychiatric interview. European Psychiatry. 1998;13:198s.
18. American Psychiatric Association. Diagnostic and statistical manual of mental disorders : DSM-5. Fifth edition. ed. Arlington, VA: American Psychiatric Association; 2013.
19. Hentati A, Forsell E, Ljótsson B, Kaldo V, Lindefors N, Kraepelien M. The effect of user interface on treatment engagement in a self-guided digital problem-solving intervention: A randomized controlled trial. Internet interventions. 2021;26:100448.
20. Salkovskis PM, Rimes KA, Warwick HMC, Clark DM. The Health Anxiety Inventory: development and validation of scales for the measurement of health anxiety and hypochondriasis. Psychological medicine. 2002;32(5):843-53.
21. Österman S, Axelsson E, Lindefors N, Hedman-Lagerlöf E, Hedman-Lagerlöf M, Kern D, et al. The 14-item short health anxiety inventory (SHAI-14) used as a screening tool: appropriate interpretation and diagnostic accuracy of the Swedish version. BMC Psychiatry. 2022;22(1):701.
22. Devilly GJ, Borkovec TD. Psychometric properties of the credibility/expectancy questionnaire. J Behav Ther Exp Psychiatry. 2000;31(2):73-86.
23. Attkisson CC, Zwick R. The client satisfaction questionnaire. Psychometric properties and correlations with service utilization and psychotherapy outcome. Eval Program Plann. 1982;5(3):233-7.
24. Titov N, Dear BF, Johnston L, Lorian C, Zou J, Wootton B, et al. Improving adherence and clinical outcomes in self-guided internet treatment for anxiety and depression: randomised controlled trial. PloS one. 2013;8(7):e62873.
25. Titov N, Dear B, Nielssen O, Staples L, Hadjistavropoulos H, Nugent M, et al. ICBT in routine care: A descriptive analysis of successful clinics in five countries. Internet interventions. 2018;26(13):108-15.

For peer review only

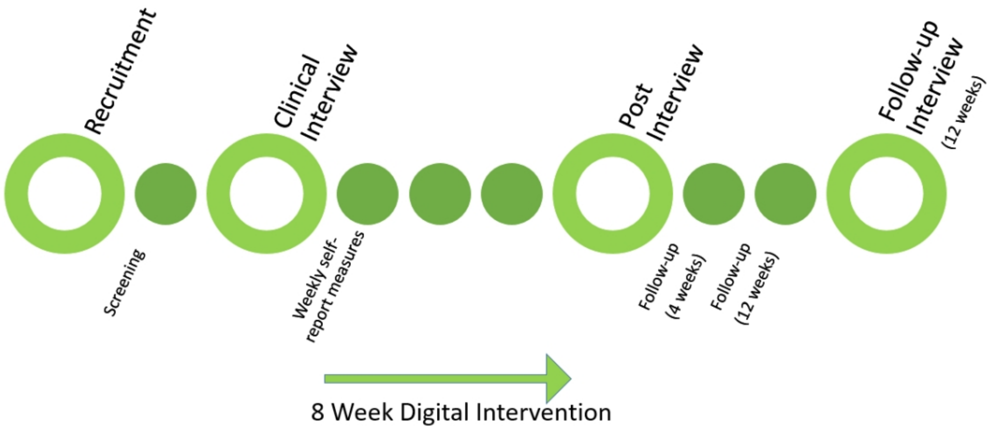

Figure 1. Flow chart of the clinical procedures

320x160mm (96 x 96 DPI)

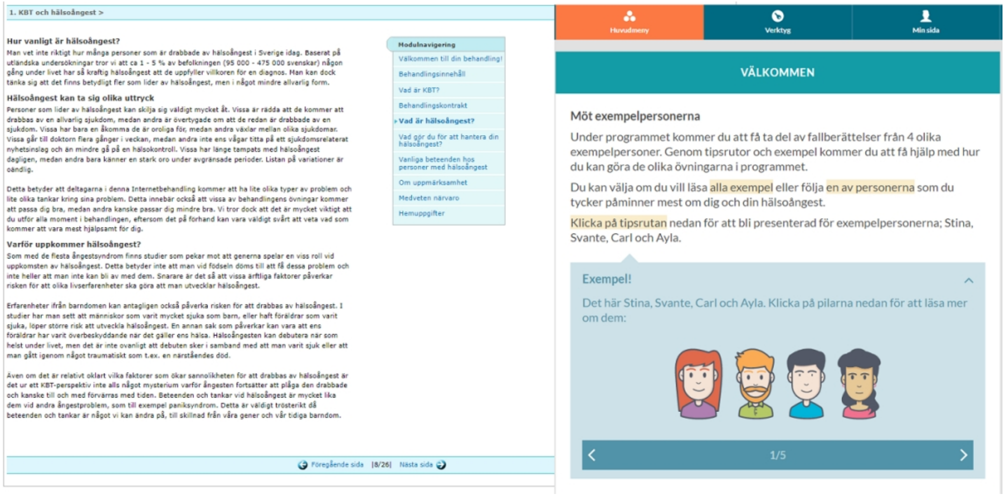

Figure 2. Screenshots from the original intervention (left) and the new self-care intervention (right).

238x117mm (144 x 144 DPI)

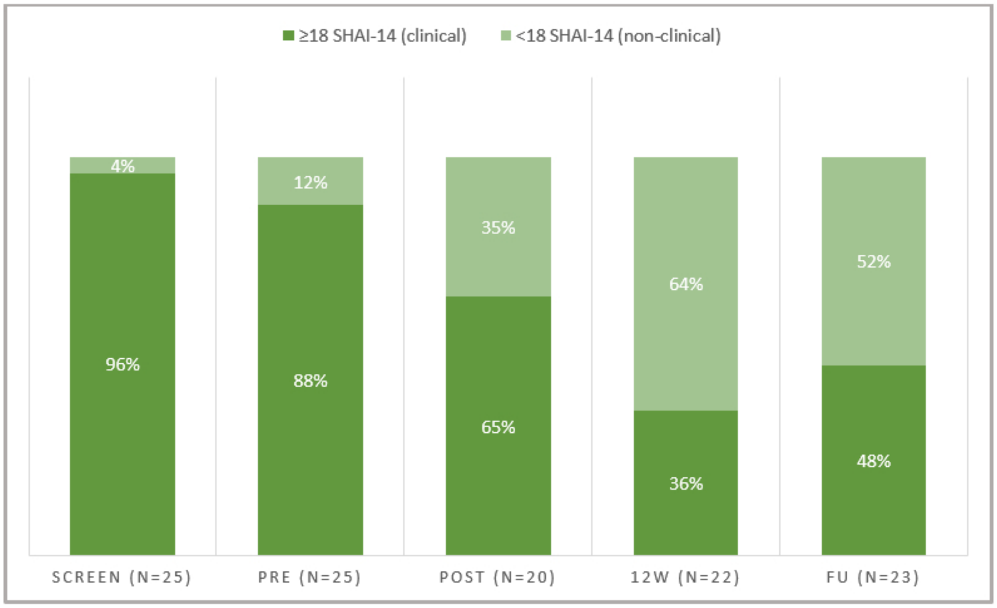

Figure 3. Proportion scoring below/above the cut-off for remission

211x131mm (96 x 96 DPI)

A feasibility study of a digital intervention for health anxiety

Supplemental material

MEAURES

Anxiety symptoms:

Generalized Anxiety Disorder 7-item scale (GAD-7) (1). The GAD-7 ranges from 0-21 where higher total scores indicate greater severity of anxiety symptoms.

Depression:

Patient Health Questionnaire 9 (PHQ-9) (2). The PHQ-9 ranges from 0 to 27 where higher total scores indicate greater severity of depression.

Quality of life:

Brunnsviken Brief Quality of Life Questionnaire (BBQ) (3). The BBQ is a 12-item questionnaire covering six different life domains: leisure time, view on life, creativity, learning, friends and friendship, and view of self. Scores range from 0 to 96, where higher values indicate higher quality of life.

Additional adherence measures:

Subjective adherence was determined by how much they had worked with ERP measured by a weekly questionnaire. In this questionnaire, participants were instructed to rate whether they had worked with ERP *not at all, occasionally, a majority of the days* or *every day* during the previous week.

Adherence was also explored by the number of different types of exposure exercises performed by participants measure by a questionnaire where participants at the end of the program was instructed to answer yes or no to whether they engaged in each component (type of exposure exercise) of the intervention.

Usability:

To assess usability, The System Usability Scale (SUS) was used (4). The SUS consists of ten items, with a range of 0-4 points per item. The items are summed up and calculated with a formula resulting in a total score between 0 and 100, with a higher score reflecting better system usability.

Experience with the program:

Experience with the self-care intervention was evaluated through a non-validated questionnaire. The questionnaire comprised four questions where participants were asked whether the intervention was perceived as likable, easy to understand, whether examples given felt relevant and whether functionality and information contributed to the participant feeling overwhelmed. The questions were answered on a four-point scale from 0 (strongly disagree) to 3 (strongly agree), with response alternatives reversed for the question regarding feeling overwhelmed. A score above 1.5 indicates positive attitudes.

RESULTS

Table e1 presents means, standard deviations and effect sizes the GAD-7, PHQ-9 and BBQ over the treatment- and follow-up period. At 3 month-follow-up, 14 out of 19 interviewed participants (74%) did not longer fulfil diagnostic criteria for IAD measured with the HPDI.

Experience with the self-care intervention:

For peer review only - http://bmjopen.bmj.com/site/about/guidelines.xhtml

A feasibility study of a digital intervention for health anxiety

The participants' responses on the study-specific questions on usefulness at post-treatment suggest that they found the intervention easy to understand (M 2.1, SD 0.72 CI 1.76-2.44) and as having relevant clinical examples (M 2.1, SD 0.83 CI 1.66-2.44). The scores on questions about the intervention being perceived as enjoyable (M 1.6, SD 0.82) and not overwhelming (M 1.75, SD 0.72 CI 1.22- 1.98) were somewhat lower.

Adherence:

In any given week during the program, 48-72% of participants reported working with ERP occasional days and 16-44 % reported working ERP a majority of the days. Moreover, 92 % of the participants reported working with ERP at least one week during the program. The mean weeks of active work with ERP was 4.6 out of 8. At post-treatment, 75 % of the participants reported that they had worked with and benefited from interoceptive exposure, 85 % from response prevention, 85 % from exposure in vivo and 70 % from imaginary exposure.

Table S1. Secondary results

Measure	Time point				Changes from PRE	
		N	M	SD	Within-group effect size	Confidence interval
GAD-7	PRE	25	8.76	4.86	NA	NA
	POST	20	5.4	4.56	0.70	0.09-1.30
	FU	22	4	3.73	1.07	0.46-1.68
PHQ-9	PRE	25	6.56	6.10	NA	NA
	POST	20	3.65	3.65	0.56	-0.04-1.15
	FU	22	3.45	4.24	0.58	-0.01-1.16
BBQ	PRE	25	52.16	19.20	NA	NA
	POST	20	58.7	17.45	0.35	-0.24-0.94
	FU	22	61.86	22.40	0.46	-0.12-1.04
SUS	Post		75.1	14.9	NA	NA

Note. Abbreviations: *GAD-7:* Generalized Anxiety Disorder 7-item scale; *PHQ-9* Patient Health Questionnaire - 9 Item; *BBQ:* Brunnsviken Brief Quality of Life Questionnaire; *SUS:* The System Usability Scale; *HPDI*: The Health Preoccupation Diagnostic Interview. *FU:* Follow up, refers to 3 months after post-treatment.

For peer review only

A feasibility study of a digital intervention for health anxiety

Supplementary references:

1. Spitzer RL, Kroenke K, Williams JBW, Löwe B. A Brief Measure for Assessing Generalized Anxiety Disorder: The GAD-7. Archives of Internal Medicine. 2006;166(10):1092-7.
2. Kroenke K, Spitzer RL, Williams JB. The PHQ-9: validity of a brief depression severity measure. J Gen Intern Med. 2001;16(9):606-13.
3. Lindner P, Frykheden O, Forsström D, Andersson E, Ljótsson B, Hedman E, et al. The Brunnsviken Brief Quality of Life Scale (BBQ): Development and Psychometric Evaluation. Cognitive behaviour therapy. 2016;45(3):182-95.
4. Bangor A, Kortum PT, Miller JT. An Empirical Evaluation of the System Usability Scale. International Journal of Human–Computer Interaction. 2008;24(6):574-94.

For peer review only

For peer review only - http://bmjopen.bmj.com/site/about/guidelines.xhtml

A Brief Digital Self-Care Intervention for Health Anxiety in a Swedish Medical University Clinic: A Prospective Single-Group Feasibility Study

Journal:	*BMJ Open*
Manuscript ID	bmjopen-2023-077376.R1
Article Type:	Original research
Date Submitted by the Author:	13-Nov-2023
Complete List of Authors:	Österman, Susanna; Karolinska Institute, Department of clinical neuroscience<br>Hentati, Amira; Karolinska Institute, Department of clinical neuroscience<br>Forsell, Erik; Karolinska Institute, Department of clinical neuroscience<br>Axelsson, Erland; Karolinska Institute, Division of Family Medicine and Primary Care, Department of Neurobiology, Care Sciences and Society<br>Hedman-Lagerlöf, Erik ; Karolinska Institute, Department of clinical neuroscience<br>Lindefors, Nils; Karolinska Institute, Department of clinical neuroscience<br>Ivanov, Volen; Karolinska Institute, Department of clinical neuroscience<br>Kraepelien, Martin; Karolinska Institute, Department of clinical neuroscience
**Primary Subject Heading**:	Mental health
Secondary Subject Heading:	Mental health
Keywords:	MENTAL HEALTH, PSYCHIATRY, Anxiety disorders < PSYCHIATRY

SCHOLARONE™
Manuscripts

I, the Submitting Author has the right to grant and does grant on behalf of all authors of the Work (as defined in the below author licence), an exclusive licence and/or a non-exclusive licence for contributions from authors who are: i) UK Crown employees; ii) where BMJ has agreed a CC-BY licence shall apply, and/or iii) in accordance with the terms applicable for US Federal Government officers or employees acting as part of their official duties; on a worldwide, perpetual, irrevocable, royalty-free basis to BMJ Publishing Group Ltd ("BMJ") its licensees and where the relevant Journal is co-owned by BMJ to the co-owners of the Journal, to publish the Work in this journal and any other BMJ products and to exploit all rights, as set out in our licence.

The Submitting Author accepts and understands that any supply made under these terms is made by BMJ to the Submitting Author unless you are acting as an employee on behalf of your employer or a postgraduate student of an affiliated institution which is paying any applicable article publishing charge ("APC") for Open Access articles. Where the Submitting Author wishes to make the Work available on an Open Access basis (and intends to pay the relevant APC), the terms of reuse of such Open Access shall be governed by a Creative Commons licence – details of these licences and which Creative Commons licence will apply to this Work are set out in our licence referred to above.

Other than as permitted in any relevant BMJ Author's Self Archiving Policies, I confirm this Work has not been accepted for publication elsewhere, is not being considered for publication elsewhere and does not duplicate material already published. I confirm all authors consent to publication of this Work and authorise the granting of this licence.

For peer review only

A Brief Digital Self-Care Intervention for Health Anxiety: A Feasibility Study

A Brief Digital Self-Care Intervention for Health Anxiety in a Swedish Medical University Clinic: A Prospective Single-Group Feasibility Study

Word count (main text): 2428

Key words: Health anxiety; illness anxiety disorder; somatic symptom disorder, cognitive behavioral therapy; digital intervention; self-care; self-guided

For peer review only

For peer review only - http://bmjopen.bmj.com/site/about/guidelines.xhtml

A Brief Digital Self-Care Intervention for Health Anxiety: A Feasibility Study

Abstract

Objectives:

In routine psychiatric care in Stockholm, Sweden, a comprehensive therapist-guided intervention for clinically significant health anxiety is implemented. However, there is a need for more easily accessible self-care interventions to improve treatment dissemination. This study aimed to transform an existing therapist-guided digital intervention into a self-care intervention, reducing patient burden and utilized clinical resources while maintaining quality and safety.

Design:

An uncontrolled feasibility study

Setting:

Conducted at Karolinska Institutet, a medical university in Sweden, with nationwide recruitment trough online advertisements.

Participants:

Twenty-five adults utilized the self-care intervention and underwent telephone assessments, along with completing self-rated questionnaires.

Intervention:

The newly developed 8-week self-care intervention was designed to be user-friendly without therapist guidance, and to facilitate high levels of behavioural engagement.

Primary and secondary outcome measures:

Indicators of quality and safety, including changes in health anxiety severity (primary), clinician time, participant adherence, perceived credibility/satisfaction with the intervention

For peer review only - http://bmjopen.bmj.com/site/about/guidelines.xhtml

For peer review only

and adverse events, were benchmarked against a previous study of the more comprehensive intervention it was based on.

Results:

Compared to the original guided intervention, the self-care intervention was condensed in terms of text (up to 70% less reading), duration (8 weeks instead of 12), and number of exercises. Quality indicators were similar to the original version. Most participants worked actively with core components in the self-care intervention. Within-group effects on health anxiety from pre-treatment to the 3-month follow-up were large (g = 1.37; 95% CI, 0.74-2.00). No serious adverse events were reported.

Conclusions:

This brief digital self-care intervention shows potential for increasing access to treatment for individuals with health anxiety, while reducing burden on patients and clinical resources. Future studies should investigate the optimal type of intervention and support for different individuals, and if non-inferiority can be established.

Trial registration:

ClinicalTrials.gov (NCT05446766)

For peer review only - http://bmjopen.bmj.com/site/about/guidelines.xhtml

A Brief Digital Self-Care Intervention for Health Anxiety: A Feasibility Study

Strengths and limitations of this study

- Strengths of the study included the thorough diagnostic assessment and the low attrition rates at follow-up.
- The results were benchmarked against a previously published randomized trial.
- The lack of a control group as well as the small study sample limits the possibility to make causal inferences and calculate precise estimates of change.
- All participants were self-referred and, hence, particularly motivated for treatment, which may have influenced the results.

For peer review only

For peer review only - http://bmjopen.bmj.com/site/about/guidelines.xhtml

A Brief Digital Self-Care Intervention for Health Anxiety: A Feasibility Study

INTRODUCTION

Clinically significant health anxiety is a psychiatric condition characterized by an excessive fear of, or preoccupation with, having or developing a serious medical illness (1). Individuals who suffer from clinically significant health anxiety tend to report high distress, disability and increased health service utilization (2). Therefore, the development of accessible, evidence-based interventions for this debilitating condition is a major challenge for health care today.

Clinically significant health anxiety can be effectively treated with therapist-guided internet-based cognitive behavioural therapy (ICBT)(3), a format which requires less therapist support per patient compared to traditional face-to-face Cognitive Behavioral Therapy (CBT), thus making the treatment more cost effective (4-6). In order to further increase the clinical utility of CBT, the development of programs without therapist support, i.e. self-guided or self-care interventions, represent an attractive avenue because of their more immediate scalability. Self-care interventions may also reduce stigma, thereby decreasing barriers to seeking treatment (7). Furthermore, data indicate that many individuals with severe health anxiety would prefer working with self-guided interventions over guided versions (8).

A recurrent finding in research highlights the challenge of maintaining comparable quality in self-help interventions, like that of guided interventions (9). However, there are promising studies suggesting that self-care interventions can be developed to a level where they can be comparable to guided ones (10). Several features have been cited as important in order to maintain effectiveness when clinician guidance is removed (11). These include a clinical interview prior to and post-intervention, monitoring of participant progress, high quality treatment material and automated features such as automatic messages prompting and reinforcing participants' work with the intervention.

In Sweden, a 12-week guided exposure-based digital intervention for health anxiety (12-14) has been successfully implemented in routine psychiatric care at the Stockholm Internet

For peer review only - http://bmjopen.bmj.com/site/about/guidelines.xhtml

psychiatry unit since 2018 (https://www.internetpsykiatri.se/en/). Exposure-based therapy, comprising the key component *exposure with response prevention* (ERP), involves gradual exposure to feared health-related situations or stimuli while refraining from compulsive health-related behaviours (15, 16).

To further meet patient needs, enhance treatment accessibility, and reduce the time and costs associated with clinician support, this comprehensive digital intervention could potentially be offered as a self-help intervention. The guided 12 week-digital intervention has, in addition to being used in a guided format, previously been used self-guided but otherwise unaltered version (14). This self-guided version showed expected and clinically significant effects but somewhat lower adherence compared to the guided intervention (around 50 % completed modules compared to 70 %). In both the guided and self-guided versions of the intervention, the key component ERP was not introduced before week 4-5 in the treatment program. This means that many individuals with low treatment adherence, especially in the unguided self-guided version, did not have sufficient time to work with this key component. Other drawbacks of the original intervention include its relative comprehensiveness, which places significant demands on the patient, and the lack of optimization in terms of user-friendliness or suitability for self-guided use.

The development of a brief self-care intervention with the goal of increasing active engagement with the main treatment components and delivering it within a structured care process, may have the potential to improve its scalability. It could decrease participant burden and clinician resources, while maintaining the inherent quality seen in therapist-guided interventions. However, before conducting a large, randomized controlled trial, an evaluation of issues related to the feasibility and acceptability of such an intervention should be conducted.

For peer review only - http://bmjopen.bmj.com/site/about/guidelines.xhtml

The aim of this study was thus to evaluate whether individuals with health anxiety find the intervention acceptable, adhere to it over time, and if it can be safely used without therapist guidance. Additionally, we aimed to determine how much clinician time such an intervention would require. Feasibility measures included participant adherence, perceived treatment credibility/satisfaction, adverse effects, and clinician time. Finally, we aimed to evaluate the preliminary efficacy of this brief intervention on symptoms of health anxiety. Measures of feasibility as well as preliminary efficacy were benchmarked to a previous study based on the more extensive and resource-intensive therapist-guided intervention it was based on (14).

MATERIAL AND METHODS

Context and design

This study was set up within the framework of a digital self-care development project initiated in 2018 and funded by the Swedish Ministry of Health and Social Affairs. The primary goal of this project is to improve the accessibility of psychological treatment in Sweden by creating concise digital self-care interventions derived from already existing digital and comprehensive therapist-guided interventions. The prospective single-group feasibility study was conducted at the Centre for Psychiatry Research, Stockholm Health Care Services, and Karolinska Institutet, Sweden. The study employed nationwide recruitment through advertisements on social media platforms. The study was preregistered on ClinicalTrials.gov (NCT05446766). Measures of feasibility and preliminary effects was benchmarked against a more comprehensive digital intervention, in both guided and unguided versions from a previously published randomised trial (14).

For peer review only - http://bmjopen.bmj.com/site/about/guidelines.xhtml

A Brief Digital Self-Care Intervention for Health Anxiety: A Feasibility Study

Patient and Public Involvement

Patients were not involved in the design of the study or the intervention. However, the research team possesses significant clinical experience with the patient group as all are clinicians/researchers. Moreover, participants underwent a telephone interview after the intervention, during which they were asked about their experience with the intervention. These comments will be utilized in future revisions of the intervention.

Participants and recruitment

For evaluation of the feasibility measures, a sample of 20 participants was deemed to be sufficient. Regarding preliminary within-group effect measured with the SHAI-14, to detect a moderate effect size (Hedges' g = 0.6), a sample of 24 participants was required, given 80% power and an α level set to $p<.05$ (2-tailed). To allow for some attrition, the goal was to recruit 25–30 participants. These were recruited through advertisements in social media under the heading “Do you worry a lot about your health?” and started the intervention after a clinical interview via telephone (average time: 40 minutes, SD: 6.9). This assessment was conducted by a clinical psychologist, and included the Health Preoccupation Diagnostic Interview (HPDI) (17) and the Mini-International Neuropsychiatric Interview (MINI) (18). Participants also underwent an interview via telephone post intervention (average time: 23 minutes, SD: 5.7). Inclusion criteria were 1) age of 18 years or older, 2) a principal diagnosis of illness anxiety disorder or somatic symptom disorder (the prototypical diagnoses for clinically significant health anxiety according to the DSM-5) (1, 19), 3) currently not receiving similar psychological treatment for health anxiety, and 4) no serious medical illness or urgent need for more intensive psychiatric care. If participants were on psychotropic medication, the dose had to be stable for 4 weeks prior to the baseline assessment. Recruitment started on September 6, 2022 and the last follow-up was collected on March, 29, 2023.

For peer review only - http://bmjopen.bmj.com/site/about/guidelines.xhtml

Intervention

The digital intervention was based on the theoretical literature regarding self-help interventions as well as clinical experience with the patient group. The graphical user interface was developed together with experts on user experience, with the aim of making the intervention easy to use without therapist guidance and to facilitate high levels of behavioural engagement (20). The self-care intervention was provided within a structured care process that included clinical interviews before and after the intervention. These interviews were considered a vital part of the intervention, ensuring diagnostic accuracy, and was intended to be a motivational tool, encouraging participants to adhere to the intervention components. Participants also received automated text messages in order to encourage engagement with the treatment. If in need of technical support, they could ask for assistance via the platform and were then contacted via telephone or email within approximately 1-3 days. For a flow chart of the clinical procedures, se Figure 1.

The content of the intervention was based on an already existing guided digital intervention for health anxiety but was adapted to be self-guided. One of the main goals was to make the intervention less demanding for the participant. This was achieved by changing aspects such as treatment length, the number of therapeutic techniques, and the complexity of the treatment material. See Table 1 for a detailed comparison between the new self-care intervention and the original comprehensive digital intervention in both therapist-guided and self-guided versions. In the new intervention, the primary therapeutic focus remained on ERP (16), a highly demanding therapeutic technique. To alleviate participant burden, extraneous elements like mindfulness training were excluded. Mindfulness was viewed not as a standalone technique but as a tool for exposure facilitation in the original intervention. In the new self-care program, ERP adherence was bolstered through early presentation, weekly problem-

For peer review only - http://bmjopen.bmj.com/site/about/guidelines.xhtml

solving sections, clinical vignettes guiding ERP challenges, and an optimized user interface. These enhancements aimed to boost participant engagement with the central ERP component.

ERP was introduced the very first week of the intervention. ERP is thought to decrease symptoms of health anxiety through repeated confrontation with feared or avoided stimuli (e.g., body sensations, situations) while refraining from engaging in maladaptive health-related behaviors (e.g., body checking) (15, 16). The intervention included both mandatory and optional text sections with the latter mainly consisting of different clinical vignettes in order to make the intervention more personalized. It also included illustrative pictures to enhance the clarity of the content, as well as expandable 'learn more' options. These features were designed to limit the amount of content displayed simultaneously and make it less demanding for the participant to absorb the material. They were also aimed to help patients differentiate between what we considered "necessary psychoeducation" and "extracurricular" texts for those who wanted more. See Figure 2 for comparative screenshots.

Table 1. Overview of the themes of the original and the new interventions including word count

	**Original digital intervention (same content in guided and self-guided version)**		**New digital self-care intervention**		
Week	Content	Word length (*n*)	Content	Word length (*n*) Mandatory	Word length (*n*) Optional
1	Introduction to CBT and health anxiety. How to navigate the program.	8528	Information about health anxiety. How to navigate the program.	5857	2493

For peer review only

A Brief Digital Self-Care Intervention for Health Anxiety: A Feasibility Study

	Introduction to mindfulness and behavior diary.		Psychoeducation about ERP		
2	Presentation of the CBT model of severe health anxiety, continued mindfulness training and behavior diary.	6558	The role of bodily symptoms. Exposure: interoceptive exposure.	2564	1248
3	Cognitive processes, continued mindfulness training.	7699	The role of safety behaviors and the importance of response prevention. Participants continue to practice ERP.	2878	3185
4	Introduction to exposure: interoceptive exposure exercises, continued mindfulness training	7961	Avoidance behavior and exposure to health anxiety provoking stimuli (In vivo exposure). Participants continue to practice ERP	1610	3059
5	Response prevention, continued mindfulness training. Participants continue to practice ERP	7454	Thoughts: Exposure to illness thoughts through imaginal exposure. Participants continue to practice ERP	2120	4730
6	Exposure to health anxiety provoking stimuli (exposure in vivo). Participants continue to practice ERP	5702	Thoughts: Exposure to illness thoughts through imaginal exposure. Participants continue to practice ERP	1418	4404
7	Imaginal exposure and exposure to the fear of death. Participants continue to practice ERP	5381	Exposure and quality of life. Participants continue to practice ERP	1555	3748
8	Continued imaginal exposure. Participants continue to practice ERP	3486	Relapse prevention: construction of a relapse plan and plan for future goals. How to handle health-care utilization in the future.	1845	2066
9	Common obstacles to exposure. Participants continue to practice ERP	1504	NA		
10	Setting new goals. Participants continue to practice ERP	396	NA		
11	A summary of the treatment. Values and	3197	NA		

For peer review only - http://bmjopen.bmj.com/site/about/guidelines.xhtml

	quality of life. Participants continue to practice ERP				
12	Maintaining gains and preventing relapse. How to handle health-care utilization in the future.	3522	NA		
Total		61388		19847	24933

Abbreviations: CBT Cognitive behaviour therapy, *ERP* Exposure with response prevention, *NA* Not applicable. Original digital intervention refers to the intervention described in Hedman et al (2016)(14)

[INSERT FIGURE 1 HERE]

[INSERT FIGURE 2 HERE]

Measures

The Health Preoccupation Diagnostic Interview (HPDI) (17) is a structured interview designed to aid the assessment of illness anxiety disorder and somatic symptom disorder according to DSM-5 criteria. The HPDI can be administered with high interrater reliability and was administered via telephone pre-treatment and at 12-week follow-up (17).

Self-report questionnaires were administered on the same technical platform where the self-care intervention was delivered, and participants received text reminders about these. Primary outcome was change in health anxiety as measured with the widely used 14-item Short Health Anxiety Inventory (SHAI-14) which has excellent psychometric properties (21) and shows evidence of good diagnostic utility in both clinical and non-clinical settings (22). Participants completed the SHAI-14 at baseline, each week during treatment, after treatment (week 8), after four additional weeks (week 12) and at the 3-month follow-up (week 24).

To assess credibility and expectancy, the five-item version of Credibility/Expectancy Questionnaire (CEQ) was administered 2 weeks after treatment start (23). A total score of 30 points or higher is considered to indicate adequate treatment credibility. At post-treatment,

For peer review only - http://bmjopen.bmj.com/site/about/guidelines.xhtml

satisfaction with the treatment was evaluated using the Client Satisfaction Questionnaire (CSQ-8) (24). To assess safety, information on possible adverse events were collected with an open-ended question at post-treatment where participants reported whether they had experienced any negative or unwanted effects of the treatment. Adherence was determined in terms of the number of completed modules, and through weekly questions about use of ERP. Additional measurements of psychiatric symptoms, quality of life and adherence are described in Supplement 1.

Analysis

Most quality indicators, including adherence, patient feedback and symptom outcomes, are reported descriptively. All analyses were based on observed data. Within-group standardized effect sizes were calculated for symptom change (Hedges *g* based on pooled SD with 95% confidence interval [CI]) at week 12 and at the 3-months follow-up. Week 12 was chosen to be able to compare the within-group effect sizes with the 12 week long interventions in the reference study (14). This comparison was with previously published data and was intended as a non-randomized benchmark. Remission was defined as scoring below 18 points on the SHAI-14 (22).

Ethical considerations

The new self-care intervention was developed based on a previously evaluated treatment protocol which has been deemed safe. Study registration was administered online on a secure platform where prospective participants provided digital informed consent and contact information. All individuals who registered to the study were contacted and informed of whether they qualified to participate. The authors declare that all procedures contributing to this work comply with the ethical standards of the relevant national and institutional committees on human experimentation and with the Helsinki Declaration of 1975, as revised

For peer review only - http://bmjopen.bmj.com/site/about/guidelines.xhtml

in 2008. All procedures involving human subjects/patients were approved by the Swedish Ethical Review Authority (identifier 2021-04133).

RESULTS

In total, 25 out of 33 interviewed participants were included and received access to the self-care intervention. Twenty-two participants (88%) provided outcome data at the week 12 follow-up, and 23 (92%) did so at the 3-month follow-up. Overall, participant demographics were comparable to those in the reference study. For participant demographics, revisions of the intervention and comparisons of quality indicators between the new self-care intervention and the reference study, see Table 2. For an overview on the proportions of participant who scored below 18 points on the SHAI-14 (criteria for remission) between time points, see Figure 3. All participants except two (23/25, 92 %) reported having worked with ERP during the treatment period. Regarding safety, three participants of those who answered the questions on adverse events (3/20, 15 %) experienced an adverse event during the treatment period. These were (i) increased anxiety, (ii) increased awareness of bodily symptoms and (iii) feelings of being alone/exposed and not being able to discuss this with a therapist. There were no reports of serious adverse events. See Supplement 1 for outcomes on additional secondary measures.

Table 2. Participant demographics, program characteristics and quality indicators benchmarked against the original interventions in the reference study

Part of treatment	Original digital intervention (guided version)	Original digital intervention (self-guided version)	New digital self-care intervention
**Demographics**			
	(*n*=32)	(*n*=33)	(*n*=25)
Gender, *n* (%)	22 (88%) female	24 (73%) female	21 (84%) female
Age<br>Mean age (SD)<br>Minimum-maximum	 38.6 (12.6)<br>22-70	 37.4 (11.6)<br>20-72	 41.0 (15.2)<br>21-70

For peer review only - http://bmjopen.bmj.com/site/about/guidelines.xhtml

For peer review only

A Brief Digital Self-Care Intervention for Health Anxiety: A Feasibility Study

Occupational status, *n* (%)			
Working full-time/part-time	22 (69%)	27 (82%)	15 (60%)
Retired, on sick-leave or unemployed	3 (9%)	3 (9%)	7 (28%)
Other	7 (22%)	4 (12%)	8 (32%)
Marital status Married or de facto, *n* (%)	28 (88%)	29 (88%)	17 (68%)
Years with severe health anxiety mean (SD)	7.2 (5.7)	7.8 (6.4)	9.0 (9.4)
Psychiatric comorbidity[a]			
Depressive disorder	1 (3 %)	11 (33%)	5 (20 %)
Anxiety disorder	14 (44%)	16 (48%)	13 (52%)
Stabilised psychotropic medication, SSRIs or SNRIs, *n* (%)	7 (22%)	7 (21%)	8 (32%)
Severity at pre SHAI-14 (0-42)	26.8 (5.4)	28.2 (6.1)	25.7 (6.1)
**Program**			
Duration	12 weeks	12 weeks	8 weeks
User interface	Desktop optimized	Desktop optimized	Mobile optimized
Number of words	60426	60426	Mandatory: 19847 Optional: 24933
Therapist time	M (SD)= 63.6 (35.4) minutes/participant	0 minutes by design	0 minutes by design
Means of support	Personalized therapist messages	None	Generalized automated text-messages
Inactivity reminders	Text-message or phone call from clinician	Text-message or phone call from clinician	Generalized automated text-messages
Unlocking new modules	Unlocked from start with instruction to complete them in consecutive order	Unlocked from start with instruction to complete them in consecutive order	Automated if/when: 1 week had passed + participant interacted with all content
**Quality indicators**			
Adherence, modules M (SD), %	8.6 (3.0), 72 %	6.6 (3.6), 55 %	5.3 (2.8), 66 %
Treatment credibility M (SD)	37.1 (6.9)	36.8 (7.9)	33.5 (13.4)

For peer review only - http://bmjopen.bmj.com/site/about/guidelines.xhtml

Completed 12 week quality indicators, *n* (%)	31 (97%)	32 (97%)	22 (88%)
Effect size (95% CI) Within-group effect on primary outcome 12 weeks [b]	1.55 (0.87-2.22)	1.31 (0.84-1.78)	1.42 (0.78-2.06)
Below cut-off (remission) at 12 weeks [b], *n* (%, 95% CI)	19, (61%, CI=42-78%)	17 (53 %, CI=35-71%)	14 (64%, CI=41-83%)
Treatment satisfaction M (SD)	26.4 (4.6)	24.8 (4.1)	23.6 (5.3)
Adverse events (at least one) *n* (%)	6 (19%)	4 (12%)	3 (14%) [c]
Completed FU quality indicators, *n* (%)	31 (97%)	27 (82%)	23 (92%)
Effect size (95% CI) Within-group effect on primary outcome FU [d]	2.23 (1.31-3.14)	1.52 (0.90-2.15)	1.37 (0.74-2.00)
Below cut-off Remission at FU [d], *n* (%, 95% CI)	20 (65 %, CI=45-81%)	17 (63%, CI=42-81%)	12 (52%, CI=28-72%)

Note [a] Psychiatric comorbidity refers to having a current DSM-5 diagnosis according to the MINI (18). "Depressive disorder" refers to major depressive disorder. "Anxiety disorder" refers to panic disorder, agoraphobia without panic disorder, social anxiety disorder, obsessive compulsive disorder, generalized anxiety disorder and post-traumatic stress disorder. [b] 12 week refers to post treatment in the reference study and 4 weeks after post treatment in the new intervention. [c] Adverse events was reported at post treatment in the present study (week 8), number of participants that completed the measurement was n=20. [d] Follow-up period (FU) refers to 6 months after post-treatment in the reference study and 3 months after post-treatment in the new intervention. Remission is defined as a score below 18 points on primary outcome. *Abbreviations: M*= Mean, *SD*=Standard deviation, *FU*= Follow-up. *SHAI-14*=14 item Short health anxiety inventory. Original digital intervention refers to the intervention described in Hedman et al (2016) (14).

[INSERT FIGURE 3 HERE]

DISCUSSION

The aim of this study was to develop and evaluate the feasibility of a newly developed brief digital self-care intervention for individuals with clinically significant health anxiety and compare it to the more comprehensive and resource-demanding digital intervention it was

For peer review only - http://bmjopen.bmj.com/site/about/guidelines.xhtml

based on. The purpose of this was to further enhance treatment accessibility for a patient population that currently presents a challenge to the healthcare system. In comparison to both the therapist-guided and self-guided version of the original intervention in the reference study, quality indicators such as participant adherence, satisfaction with treatment, preliminary within-group effect sizes as well as safety were promising. Importantly, almost all participants in the new self-care intervention worked with ERP during the treatment period.

Compared to the reference study, the participants appear to share similar demographics and disease burden and the time spent on interviews of around 60 minutes per participant was also similar (14). However, in the new self-care intervention, the overall clinician workload was reduced since no written guidance were provided which was the case in the guided original intervention where clinician on average posted 18.4 text messages (SD 9.6), mean time: 63.6 minutes (SD: 35.4). Moreover, in both the guided and the self-guided original intervention, the clinician contacted the participant via SMS or phone if they had not been active on the platform for a week, which was not the case in the new self-care intervention. This moderately high level of structure and contact may have worked as a form of engagement enhancing procedures - similar to written guidance. The relatively high level of adherence observed in the new self-care intervention, despite the absence of such contact, is therefore promising and aligns with previous studies indicating that a well-designed self-help intervention may compensate for the lack of therapist support (11, 25). Furthermore, preliminary within-group effects seemed promising given the fact that the new self-care intervention posed less burden on participants due to a shorter treatment length, less and more simplified text and a significant reduction in the number of different types of mandatory exercises. Since the guided intervention in the reference study had seemingly higher remission rates at the follow-up assessment, there may, however, be some participants who would

For peer review only - http://bmjopen.bmj.com/site/about/guidelines.xhtml

benefit from receiving additional guidance. Future studies could help identify who would benefit from such guidance.

Although preliminary, this study supports the notion that a brief digital self-care intervention is feasible and can be comparable to more comprehensive guided versions and should be further examined in larger studies. In order to maintain adherence as well as treatment effects, factors such as the self-care intervention being provided within a clinical context with a short clinical interview prior and post to the intervention are potentially important.

The study has some limitations such as its small sample size and the absence of randomization. Since the comparisons with the original comprehensive intervention in the reference study were not randomized, there was some apparent differences in population characteristics, such as levels of psychiatric comorbidity. Additionally, the recruitment methods differed between the reference study and the current study. The reference study used both advertisements and recruitment from healthcare professionals, while the current study solely relied on advertisements. This variation may have caused sample disparities. However, only 31 out of 132 participants in the reference study were recruited from a medical clinic. Further, the period between the two studies included a pandemic, potentially influencing the population's mental health awareness and levels of worry. This situation might have attracted different types of patients interested in participating in an intervention focused on health anxiety. These factors render caution in all interpretation of differences and similarities in the comparison of interventions. Nevertheless, a strength lies in the fact that all participants in both the original and current studies underwent the same thorough diagnostic assessment, the two studies use the same eligibility criteria as well as that the level of health anxiety measured with a well-validated measure appear to be similar. The current study primarily employed self-report measures. It is important to bear in mind the limitations inherent in these types of instruments, such as the reliance on the cooperation of the person answering them and the

For peer review only

potential presence of recall and response bias. A potential improvement in a future study could be to conduct diagnostic interviews at post treatment by a blind assessor and/or measure changes in healthcare consumption before and after treatment, based on patient register data Further, many adjustments from the original intervention were made, both regarding content and intervention processes. These changes have made it difficult to evaluate the feasibility of each component separately. Finally, the sample consisted of self-recruited volunteers. However, if implemented, a brief self-care intervention such as the current one may be a means to reach individuals who prefer working on their own and/or who would not otherwise seek help due to stigma or other reasons. It could also possibly serve as a first low intensity intervention in a stepped care model. Additionally, the vast majority of patients receiving ICBT in implemented regular care settings are also self-referred (26).

In conclusion, the quality indicators did not seem to show any substantial differences between the brief self-care intervention compared to the original more resource demanding intervention. This highlights the need for larger randomized studies evaluating what type of intervention works for whom, the role of guidance as well as the level of comprehensiveness from both a cost-effectiveness as well as participant- and clinician burden perspective, as well as studies of possible non-inferiority. Future research should investigate the impact of different treatment components on outcomes, as well as participants' satisfaction and adherence. A study utilizing a factorial design (27), adjusting treatment components, therapist support levels, and treatment duration, could shed light on these aspects. If this brief self-care intervention is found to be both effective and resource effective, it could be a highly useful treatment option for patients with clinically significant health anxiety.

For peer review only - http://bmjopen.bmj.com/site/about/guidelines.xhtml

A Brief Digital Self-Care Intervention for Health Anxiety: A Feasibility Study

Figure caption

Figure 1. Flow chart of the clinical procedures

Figure 2. Screenshots from the original intervention (left) and the new self-care intervention (right).

Figure 3. Proportion scoring below/above the cut-off for remission

Proportions of participants scoring below/above the cut-off for remission (18 points) on the SHAI-14 at different time points throughout the trial. Post refers to 8 weeks after treatment start. 12 W refers to 4 weeks after post-treatment (week 12). FU refers to the 3-month follow-up (week 24).

Funding

This study was supported by the Swedish Ministry of Health and Social affairs (grant number S2018/03855/FS). The ministry had no influence on study design, data collection, analysis or publication.

Data Availability Statement

Data are available upon reasonable request.

Contributorship Statement

Susanna Österman: study design, intervention design, drafting of manuscript, statistical analysis and conducting clinical interviews. Martin Kraepelien: study concept, intervention design, study design, principal investigator. Amira Hentati: conducting clinical interviews, contributions to manuscript. Erik Forsell: Study concept, intervention design, study design. Erland Axelsson: statistical analysis and contributions to manuscript. Erik Hedman-Lagerlöf: Study concept, contributions to manuscript. Nils Lindefors and Volen Ivanov: Study concept,

For peer review only - http://bmjopen.bmj.com/site/about/guidelines.xhtml

study design, contributions to manuscript. All authors: Critical review and revision of manuscript.

Competing interests

Erik Hedman-Lagerlöf and Erland Axelsson have co-authored a self-help book for pathological health anxiety centred around exposure-based cognitive behaviour therapy, and for which they receive royalties. The remaining authors report no conflicts of interest.

For peer review only

A Brief Digital Self-Care Intervention for Health Anxiety: A Feasibility Study

References:

1. Axelsson E, Hedman-Lagerlöf E. Validity and clinical utility of distinguishing between DSM-5 somatic symptom disorder and illness anxiety disorder in pathological health anxiety: Should we close the chapter? J Psychosomat Res. 2023;165:111133.
2. Fink P, Ørnbøl E, Christensen KS. The outcome of health anxiety in primary care. A two-year follow-up study on health care costs and self-rated health. PloS one. 2010;5(3):e9873.
3. Axelsson E, Hedman-Lagerlöf E. Cognitive behavior therapy for health anxiety: systematic review and meta-analysis of clinical efficacy and health economic outcomes. Expert review of pharmacoeconomics & outcomes research. 2019;19(6):663-76.
4. Hedman E, Andersson E, Ljotsson B, Axelsson E, Lekander M. Cost effectiveness of internet-based cognitive behaviour therapy and behavioural stress management for severe health anxiety. BMJ open. 2016;6(4):e009327.
5. Axelsson E, Andersson E, Ljótsson B, Björkander D, Hedman-Lagerlöf M, Hedman-Lagerlöf E. Effect of Internet vs Face-to-Face Cognitive Behavior Therapy for Health Anxiety: A Randomized Noninferiority Clinical Trial. JAMA Psychiatry. 2020;77(9):915-24.
6. Ophuis RH, Lokkerbol J, Heemskerk SCM, van Balkom AJLM, Hiligsmann M, Evers SMAA. Cost-Effectiveness of Interventions for Treating Anxiety Disorders: A Systematic Review. Journal of affective disorders. 2017;210:1-13.
7. Mills H, Mulfinger N, Raeder S, Rüsch N, Clements H, Scior K. Self-help interventions to reduce self-stigma in people with mental health problems: A systematic literature review. Psychiatry Research. 2020;284:112702.
8. Newby JM, Haskelberg H, Hobbs MJ, Mahoney AEJ, Mason E, Andrews G. The effectiveness of internet-delivered cognitive behavioural therapy for health anxiety in routine care. Journal of Affective Disorders. 2020;264:535-42.
9. Baumeister H, Reichler L, Munzinger M, Lin J. The impact of guidance on Internet-based mental health interventions—A systematic review. Internet interventions. 2014;1(4):205-15.
10. Domhardt M, Geßlein H, von Rezori RE, Baumeister H. Internet- and mobile-based interventions for anxiety disorders: A meta-analytic review of intervention components. Depression and Anxiety. 2019;36(3):213-24.
11. Titov N, Dear BF, Staples LG, Terides MD, Karin E, Sheehan J, et al. Disorder-specific versus transdiagnostic and clinician-guided versus self-guided treatment for major depressive disorder and comorbid anxiety disorders: A randomized controlled trial. Journal of anxiety disorders. 2015;35:88-102.
12. Hedman E, Andersson G, Andersson E, Ljotsson B, Ruck C, Asmundson GJ, et al. Internet-based cognitive-behavioural therapy for severe health anxiety: randomised controlled trial. The British journal of psychiatry : the journal of mental science. 2011;198(3):230-6.
13. Hedman E, Axelsson E, Gorling A, Ritzman C, Ronnheden M, El Alaoui S, et al. Internet-delivered exposure-based cognitive-behavioural therapy and behavioural stress management for severe health anxiety: randomised controlled trial. The British journal of psychiatry : the journal of mental science. 2014;205(4):307-14.
14. Hedman E, Axelsson E, Andersson E, Lekander M, Ljótsson B. Exposure-based cognitive-behavioural therapy via the internet and as bibliotherapy for somatic symptom disorder and illness anxiety disorder: randomised controlled trial. The British journal of psychiatry : the journal of mental science. 2016;209(5):407-13.
15. Visser S, Bouman TK. The treatment of hypochondriasis: exposure plus response prevention vs cognitive therapy. Behav Res Ther. 2001;39(4):423-42.
16. Furer P, Walker JR. Treatment of hypochondriasis with exposure. Journal of Contemporary Psychotherapy. 2005;35(3):251-67.

For peer review only - http://bmjopen.bmj.com/site/about/guidelines.xhtml

17. Axelsson E, Andersson E, Ljotsson B, Wallhed Finn D, Hedman E. The health preoccupation diagnostic interview: inter-rater reliability of a structured interview for diagnostic assessment of DSM-5 somatic symptom disorder and illness anxiety disorder. Cognitive behaviour therapy. 2016;45(4):259-69.
18. Lecrubier Y, Sheehan D, Hergueta T, Weiller E. SEC55-1 - The mini international neuropsychiatric interview. European Psychiatry. 1998;13:198s.
19. American Psychiatric Association. Diagnostic and statistical manual of mental disorders : DSM-5. Fifth edition. ed. Arlington, VA: American Psychiatric Association; 2013.
20. Hentati A, Forsell E, Ljótsson B, Kaldo V, Lindefors N, Kraepelien M. The effect of user interface on treatment engagement in a self-guided digital problem-solving intervention: A randomized controlled trial. Internet interventions. 2021;26:100448.
21. Salkovskis PM, Rimes KA, Warwick HMC, Clark DM. The Health Anxiety Inventory: development and validation of scales for the measurement of health anxiety and hypochondriasis. Psychological medicine. 2002;32(5):843-53.
22. Österman S, Axelsson E, Lindefors N, Hedman-Lagerlöf E, Hedman-Lagerlöf M, Kern D, et al. The 14-item short health anxiety inventory (SHAI-14) used as a screening tool: appropriate interpretation and diagnostic accuracy of the Swedish version. BMC Psychiatry. 2022;22(1):701.
23. Devilly GJ, Borkovec TD. Psychometric properties of the credibility/expectancy questionnaire. J Behav Ther Exp Psychiatry. 2000;31(2):73-86.
24. Attkisson CC, Zwick R. The client satisfaction questionnaire. Psychometric properties and correlations with service utilization and psychotherapy outcome. Eval Program Plann. 1982;5(3):233-7.
25. Titov N, Dear BF, Johnston L, Lorian C, Zou J, Wootton B, et al. Improving adherence and clinical outcomes in self-guided internet treatment for anxiety and depression: randomised controlled trial. PloS one. 2013;8(7):e62873.
26. Titov N, Dear B, Nielssen O, Staples L, Hadjistavropoulos H, Nugent M, et al. ICBT in routine care: A descriptive analysis of successful clinics in five countries. Internet interventions. 2018;26(13):108-15.
27. Watkins ER, Newbold A. Factorial Designs Help to Understand How Psychological Therapy Works. Frontiers in Psychiatry. 2020;11.

For peer review only

For peer review only - http://bmjopen.bmj.com/site/about/guidelines.xhtml

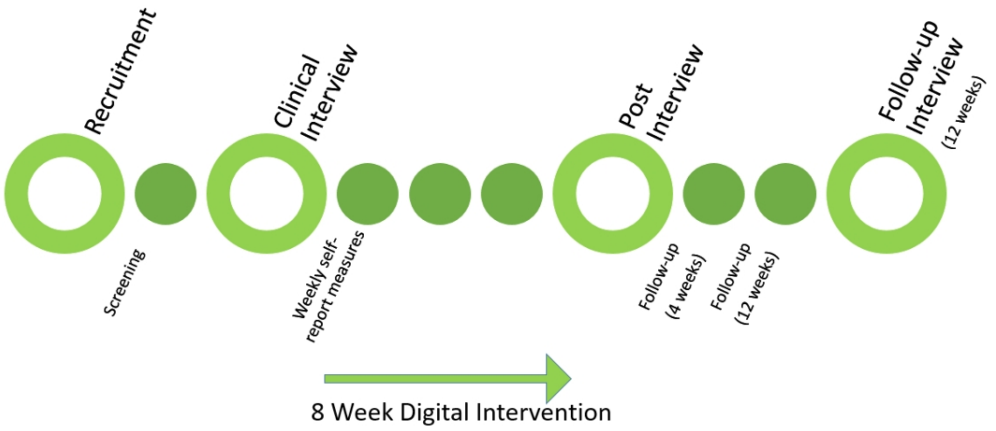

Figure 1. Flow chart of the clinical procedures

320x160mm (96 x 96 DPI)

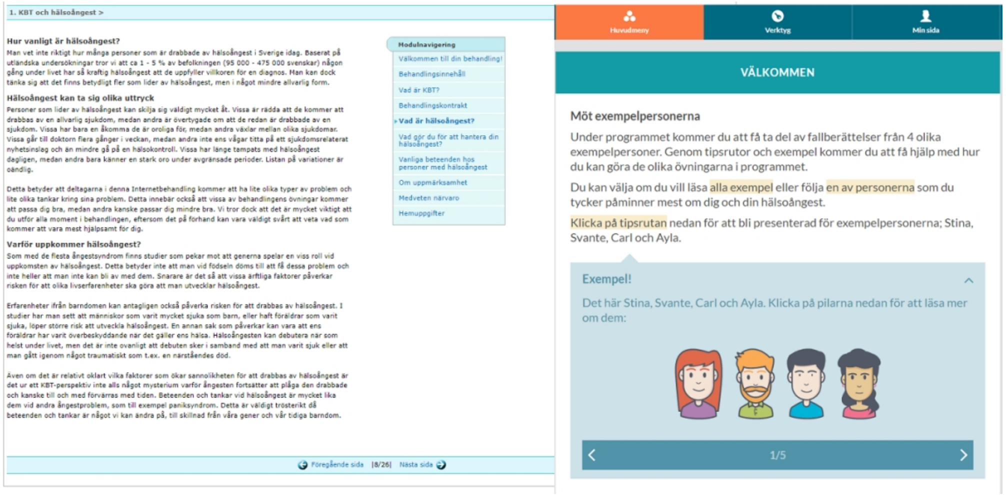

Figure 2. Screenshots from the original intervention (left) and the new self-care intervention (right).

238x117mm (144 x 144 DPI)

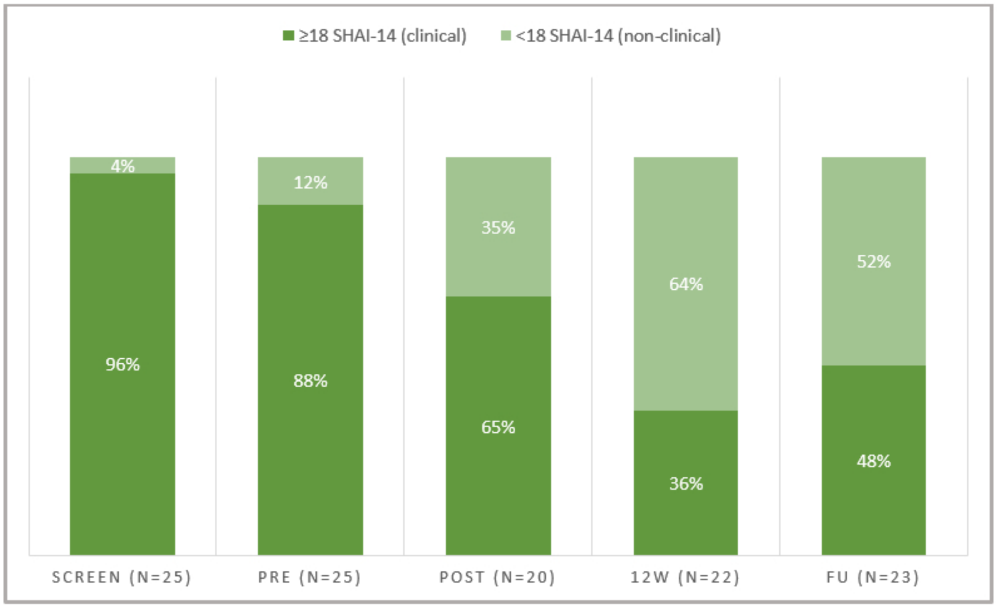

Figure 3. Proportion scoring below/above the cut-off for remission

211x131mm (96 x 96 DPI)

A feasibility study of a digital intervention for health anxiety

Supplemental material

MEAURES

Anxiety symptoms:

Generalized Anxiety Disorder 7-item scale (GAD-7) (1). The GAD-7 ranges from 0-21 where higher total scores indicate greater severity of anxiety symptoms.

Depression:

Patient Health Questionnaire 9 (PHQ-9) (2). The PHQ-9 ranges from 0 to 27 where higher total scores indicate greater severity of depression.

Quality of life:

Brunnsviken Brief Quality of Life Questionnaire (BBQ) (3). The BBQ is a 12-item questionnaire covering six different life domains: leisure time, view on life, creativity, learning, friends and friendship, and view of self. Scores range from 0 to 96, where higher values indicate higher quality of life.

Additional adherence measures:

Subjective adherence was determined by how much they had worked with ERP measured by a weekly questionnaire. In this questionnaire, participants were instructed to rate whether they had worked with ERP *not at all, occasionally, a majority of the days* or *every day* during the previous week.

Adherence was also explored by the number of different types of exposure exercises performed by participants measure by a questionnaire where participants at the end of the program was instructed to answer yes or no to whether they engaged in each component (type of exposure exercise) of the intervention.

Usability:

To assess usability, The System Usability Scale (SUS) was used (4). The SUS consists of ten items, with a range of 0-4 points per item. The items are summed up and calculated with a formula resulting in a total score between 0 and 100, with a higher score reflecting better system usability.

Experience with the program:

Experience with the self-care intervention was evaluated through a non-validated questionnaire. The questionnaire comprised four questions where participants were asked whether the intervention was perceived as likable, easy to understand, whether examples given felt relevant and whether functionality and information contributed to the participant feeling overwhelmed. The questions were answered on a four-point scale from 0 (strongly disagree) to 3 (strongly agree), with response alternatives reversed for the question regarding feeling overwhelmed. A score above 1.5 indicates positive attitudes.

RESULTS

Table e1 presents means, standard deviations and effect sizes the GAD-7, PHQ-9 and BBQ over the treatment- and follow-up period. At 3 month-follow-up, 14 out of 19 interviewed participants (74%) did not longer fulfil diagnostic criteria for IAD measured with the HPDI.

Experience with the self-care intervention:

For peer review only - http://bmjopen.bmj.com/site/about/guidelines.xhtml

The participants' responses on the study-specific questions on usefulness at post-treatment suggest that they found the intervention easy to understand (M 2.1, SD 0.72 CI 1.76-2.44) and as having relevant clinical examples (M 2.1, SD 0.83 CI 1.66-2.44). The scores on questions about the intervention being perceived as enjoyable (M 1.6, SD 0.82) and not overwhelming (M 1.75, SD 0.72 CI 1.22- 1.98) were somewhat lower.

Adherence:

In any given week during the program, 48-72% of participants reported working with ERP occasional days and 16-44 % reported working ERP a majority of the days. Moreover, 92 % of the participants reported working with ERP at least one week during the program. The mean weeks of active work with ERP was 4.6 out of 8. At post-treatment, 75 % of the participants reported that they had worked with and benefited from interoceptive exposure, 85 % from response prevention, 85 % from exposure in vivo and 70 % from imaginary exposure.

Table S1. Secondary results

Measure	Time point				Changes from PRE	
		N	M	SD	Within-group effect size	Confidence interval
GAD-7	PRE	25	8.76	4.86	NA	NA
	POST	20	5.4	4.56	0.70	0.09-1.30
	FU	22	4	3.73	1.07	0.46-1.68
PHQ-9	PRE	25	6.56	6.10	NA	NA
	POST	20	3.65	3.65	0.56	-0.04-1.15
	FU	22	3.45	4.24	0.58	-0.01-1.16
BBQ	PRE	25	52.16	19.20	NA	NA
	POST	20	58.7	17.45	0.35	-0.24-0.94
	FU	22	61.86	22.40	0.46	-0.12-1.04
SUS	Post		75.1	14.9	NA	NA

Note. Abbreviations: *GAD-7:* Generalized Anxiety Disorder 7-item scale; *PHQ-9* Patient Health Questionnaire - 9 Item; *BBQ:* Brunnsviken Brief Quality of Life Questionnaire; *SUS:* The System Usability Scale; *HPDI*: The Health Preoccupation Diagnostic Interview. *FU:* Follow up, refers to 3 months after post-treatment.

For peer review only

A feasibility study of a digital intervention for health anxiety

Supplementary references:

1. Spitzer RL, Kroenke K, Williams JBW, Löwe B. A Brief Measure for Assessing Generalized Anxiety Disorder: The GAD-7. Archives of Internal Medicine. 2006;166(10):1092-7.
2. Kroenke K, Spitzer RL, Williams JB. The PHQ-9: validity of a brief depression severity measure. J Gen Intern Med. 2001;16(9):606-13.
3. Lindner P, Frykheden O, Forsström D, Andersson E, Ljótsson B, Hedman E, et al. The Brunnsviken Brief Quality of Life Scale (BBQ): Development and Psychometric Evaluation. Cognitive behaviour therapy. 2016;45(3):182-95.
4. Bangor A, Kortum PT, Miller JT. An Empirical Evaluation of the System Usability Scale. International Journal of Human–Computer Interaction. 2008;24(6):574-94.

For peer review only

CONSORT 2010 checklist of information to include when reporting a pilot or feasibility trial*

Section/Topic	Item No	Checklist item	Reported on page No
**Title and abstract**			
	1a	Identification as a pilot or feasibility randomised trial in the title	1
	1b	Structured summary of pilot trial design, methods, results, and conclusions (for specific guidance see CONSORT abstract extension for pilot trials)	2,3
**Introduction**			
Background and objectives	2a	Scientific background and explanation of rationale for future definitive trial, and reasons for randomised pilot trial	5,6
	2b	Specific objectives or research questions for pilot trial	7
**Methods**			
Trial design	3a	Description of pilot trial design (such as parallel, factorial) including allocation ratio	7
	3b	Important changes to methods after pilot trial commencement (such as eligibility criteria), with reasons	N/A
Participants	4a	Eligibility criteria for participants	8
	4b	Settings and locations where the data were collected	7,12
	4c	How participants were identified and consented	
Interventions	5	The interventions for each group with sufficient details to allow replication, including how and when they were actually administered	8,9,10
Outcomes	6a	Completely defined prespecified assessments or measurements to address each pilot trial objective specified in 2b, including how and when they were assessed	12,13
	6b	Any changes to pilot trial assessments or measurements after the pilot trial commenced, with reasons	N/A
	6c	If applicable, prespecified criteria used to judge whether, or how, to proceed with future definitive trial	N/A
Sample size	7a	Rationale for numbers in the pilot trial	8
	7b	When applicable, explanation of any interim analyses and stopping guidelines	N/A
Randomisation:			
Sequence generation	8a	Method used to generate the random allocation sequence	N/A
	8b	Type of randomisation(s); details of any restriction (such as blocking and block size)	N/A
Allocation concealment mechanism	9	Mechanism used to implement the random allocation sequence (such as sequentially numbered containers), describing any steps taken to conceal the sequence until interventions were assigned	N/A

For peer review only - http://bmjopen.bmj.com/site/about/guidelines.xhtml

Implementation	10	Who generated the random allocation sequence, who enrolled participants, and who assigned participants to interventions	N/A
Blinding	11a	If done, who was blinded after assignment to interventions (for example, participants, care providers, those assessing outcomes) and how	N/A
	11b	If relevant, description of the similarity of interventions	N/A
Statistical methods	12	Methods used to address each pilot trial objective whether qualitative or quantitative	13
**Results**			
Participant flow (a diagram is strongly recommended)	13a	For each group, the numbers of participants who were approached and/or assessed for eligibility, randomly assigned, received intended treatment, and were assessed for each objective	14,16
	13b	For each group, losses and exclusions after randomisation, together with reasons	N/A
Recruitment	14a	Dates defining the periods of recruitment and follow-up	8
	14b	Why the pilot trial ended or was stopped	N/A
Baseline data	15	A table showing baseline demographic and clinical characteristics for each group	14,15
Numbers analysed	16	For each objective, number of participants (denominator) included in each analysis. If relevant, these numbers should be by randomised group	14,16
Outcomes and estimation	17	For each objective, results including expressions of uncertainty (such as 95% confidence interval) for any estimates. If relevant, these results should be by randomised group	16
Ancillary analyses	18	Results of any other analyses performed that could be used to inform the future definitive trial	N/A
Harms	19	All important harms or unintended effects in each group (for specific guidance see CONSORT for harms)	14
	19a	If relevant, other important unintended consequences	N/A
**Discussion**			
Limitations	20	Pilot trial limitations, addressing sources of potential bias and remaining uncertainty about feasibility	18,19
Generalisability	21	Generalisability (applicability) of pilot trial methods and findings to future definitive trial and other studies	18,19
Interpretation	22	Interpretation consistent with pilot trial objectives and findings, balancing potential benefits and harms, and considering other relevant evidence	16,17
	22a	Implications for progression from pilot to future definitive trial, including any proposed amendments	19
**Other information**			
Registration	23	Registration number for pilot trial and name of trial registry	7
Protocol	24	Where the pilot trial protocol can be accessed, if available	7
Funding	25	Sources of funding and other support (such as supply of drugs), role of funders	20
	26	Ethical approval or approval by research review committee, confirmed with reference number	13,14

For peer review only

For peer review only - http://bmjopen.bmj.com/site/about/guidelines.xhtml

Citation: Eldridge SM, Chan CL, Campbell MJ, Bond CM, Hopewell S, Thabane L, et al. CONSORT 2010 statement: extension to randomised pilot and feasibility trials. BMJ. 2016;355. This is an Open Access article distributed in accordance with the terms of the Creative Commons Attribution (CC BY 3.0) license (http://creativecommons.org/licenses/by/3.0/), which permits others to distribute, remix, adapt and build upon this work, for commercial use, provided the original work is properly cited.

*We strongly recommend reading this statement in conjunction with the CONSORT 2010, extension to randomised pilot and feasibility trials, Explanation and Elaboration for important clarifications on all the items. If relevant, we also recommend reading CONSORT extensions for cluster randomised trials, non-inferiority and equivalence trials, non-pharmacological treatments, herbal interventions, and pragmatic trials. Additional extensions are forthcoming: for those and for up-to-date references relevant to this checklist, see www.consort-statement.org.

For peer review only
